# Menthol–thymol NADES as a fungicidal and chemosensitizing agent against multidrug-resistant *Candida albicans*: ROS induction, efflux pump inhibition, and synergy with fluconazole

**DOI:** 10.3389/fphar.2025.1643472

**Published:** 2025-08-15

**Authors:** Melisa Fabiana Negro, Pamela Soledad Bustos, Lautaro Bellezze, María Gabriela Ortega, Javier Echeverría, María Fernanda Silva, Mariana Andrea Peralta

**Affiliations:** ^1^ Farmacognosia, Departamento de Ciencias Farmacéuticas, Facultad de Ciencias Químicas, Universidad Nacional de Córdoba, Córdoba, Argentina; ^2^ Unidad de Investigación y Desarrollo en Tecnología Farmacéutica (UNITEFA-CONICET), Ciudad Universitaria, Córdoba, Argentina; ^3^ Departamento de Ciencias del Ambiente, Facultad de Química y Biología, Universidad de Santiago de Chile, Santiago, Chile; ^4^ Grupo de Química Analítica Verde (GQAV), Instituto de Biología Agrícola de Mendoza (IBAM-CONICET). Facultad de Ciencias Agrarias-Universidad Nacional de Cuyo (FCA-UNCuyo), Mendoza, Argentina

**Keywords:** ecopharmacognosy, natural deep eutectic solvents, terpenes, antifungal, multidrug resistance, *Candida albicans*, efflux pump inhibition, chemosensitizer

## Abstract

**Background:**

The increasing prevalence of azole-resistant *Candida albicans* (RCa) poses a critical therapeutic challenge, necessitating innovative antifungal approaches. Natural deep eutectic solvents (NADES), derived from natural metabolites such as terpenes, provide a promising and sustainable platform for delivering bioactive compounds with intrinsic pharmacological properties.

**Purpose:**

This study evaluated a eutectic system composed of menthol and thymol (MT NADES, 1:1 M ratio) for its antifungal efficacy against a multidrug-resistant clinical *C. albicans* strain.

**Materials and methods:**

The antifungal activity of MT NADES was evaluated against a clinical *C. albicans* strain resistant to azole antifungals (RCa). The minimum inhibitory concentration (MIC) and minimum fungicidal concentration (MFC) were determined using the broth microdilution method, following the CLSI M27-A4 guidelines. Synergistic effects with fluconazole were assessed through checkerboard microdilution and disc diffusion assays, with the fractional inhibitory concentration index (FICI) calculated to quantify interactions. Intracellular reactive oxygen species (ROS) levels were quantified using DCFH-DA staining and fluorescence spectrophotometry. Efflux pump inhibition was investigated via Nile red accumulation assay, analyzed by flow cytometry, using tacrolimus (100 µM) as a positive control. All experiments were performed in triplicate.

**Results and discussion:**

MT NADES demonstrated potent fungicidal activity against resistant *C*. *albicans* with an MIC of 180 μg/mL and MFC of 360 μg/mL (MFC/MIC = 2), outperforming its components (menthol: 1000 μg/mL; thymol: 200 μg/mL). Synergistic interaction with fluconazole (MIC: 32 μg/mL) was confirmed by checkerboard and disc diffusion assays (FICI: 0.2839). Mechanistic studies revealed increased intracellular ROS, supporting oxidative stress as a key antifungal mechanism. Additionally, MT NADES at half its MIC enhanced Nile red retention 10-fold over the efflux pump inhibitor tacrolimus (100 µM), indicating strong inhibition of multidrug resistance (MDR)-related transporters. These findings highlight MT NADES as a promising chemosensitizing agent with superior efficacy over its individual components.

**Conclusion:**

These findings underscore the therapeutic potential of menthol–thymol NADES as a multifunctional, plant-derived antifungal strategy capable of overcoming multidrug resistance mechanisms and potentiating azole efficacy in *C. albicans*.

## 1 Introduction

Oral candidiasis is a high-frequency infectious disease that affects the oral cavity. It is characterized by an excessive growth of *Candida* fungi, mainly *Candida albicans*, in the oral mucosa. This fungal proliferation occurs when the host’s physical barriers and defenses are altered.

Currently, azole antifungal agents are the first choice for treating candidiasis; however, they are associated with numerous adverse effects that limit the dose and dosing frequency. Furthermore, the extensive use of antifungal agents, mainly azoles, has led to the emergence of resistant strains (Espino et al., 2019; Tan et al., 2023).

*C. albicans*’ resistance to antifungals is a major public health problem worldwide. In 2022, the World Health Organization (WHO) recognized the seriousness of this situation by declaring this pathogenic fungus a priority for the research and development of new therapeutic alternatives; thus, the treatment of this pathogen represents not only a great challenge but also an urgent need to search for new medications (WHO, 2022).

One of the key mechanisms underlying antifungal resistance in *C. albicans* is the overexpression of multidrug resistance (MDR) efflux pumps, which actively expel a wide range of antifungal agents from the cell, thereby reducing their intracellular accumulation and therapeutic efficacy. These transporters, particularly those belonging to the ATP-binding cassette (ABC) family, such as Cdr1p and Cdr2p, and to the major facilitator superfamily (MFS), such as Mdr1p, are central to the development of multidrug resistance phenotypes. MDR in *C. albicans* is increasingly associated with persistent and recurrent infections, especially in immunocompromised patients, and it severely limits the effectiveness of frontline antifungals like azoles (Garvey et al., 2022). As highlighted in recent studies, targeting these efflux mechanisms with natural chemosensitizing agents emerges as a promising strategy to overcome resistance and restore antifungal susceptibility. Understanding and disrupting MDR systems are thus pivotal in developing new therapeutic approaches against resistant fungal pathogens (Santi et al., 2022).

The continuous exploration of plant-derived compounds has led to the identification of numerous antimicrobial agents with novel mechanisms of action (Cushnie et al., 2020). Recent phytochemical studies on traditional herbal medicines have revealed over 150 new natural products with bioactive potential, many of which are components of essential oils, including monoterpenes and phenolic compounds (Tanaka and Kashiwada, 2021). These findings underscore the relevance of natural molecules such as menthol and thymol, major constituents of *Mentha* spp. and *Thymus vulgaris* essential oils, as promising candidates in the development of innovative antifungal therapies.

**FIGURE 1:**
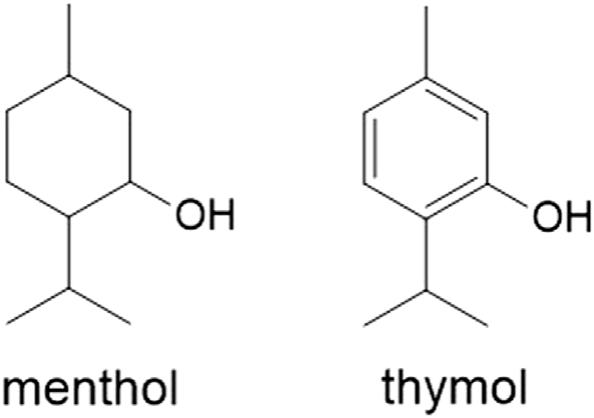
Menthol and thymol structure.

Menthol and thymol are natural terpene-derived compounds with diverse pharmacological properties and have been utilized as components in terpene-based natural deep eutectic solvents (NADES). Menthol (Men) (Figure 1), a cyclic monoterpenoid and the principal compound in *Mentha* essential oils, is well-known for its characteristic cooling aroma and exhibits antibacterial, antifungal, antiviral, analgesic, and antipruritic properties. Thymol (Thy) (Figure 1), the predominant phenolic component of *T. vulgaris* (thyme) essential oil, also demonstrates strong antimicrobial and antifungal activity. However, the pharmaceutical application of these compounds is challenged by their physicochemical limitations—menthol’s high volatility, low aqueous solubility, and tendency to crystallize, and thymol’s poor water solubility and limited miscibility in aqueous systems (Silva et al., 2021). The highlight of the development of antimicrobial formulations containing Thy and Men is that both compounds are considered “generally recognized as safe” (GRAS) by the Environmental Protection Agency (EPA).

Recent approaches in natural antimicrobial product development have focused on the application of NADES. These solvents are composed of combinations of primary and secondary cellular metabolites, such as sugars, organic acids, bases, terpenes, and amino acids, in defined molar ratios. The resulting eutectic mixtures exhibit significantly reduced melting points relative to their individual components. The structural integrity of NADES is governed by intermolecular interactions among its constituents (Mammana et al., 2023). Compared to traditional solvents, NADES are environmentally friendly—biodegradable, low in toxicity, synthesized with minimal energy input, and free from toxic waste. They are also biocompatible and non-volatile, offering efficient solubilization of bioactive compounds, thereby representing a sustainable and safer alternative for pharmaceutical and extraction processes (Hikmawanti et al., 2021; Bergua et al., 2022). Given these advantages, NADES are increasingly applied across diverse sectors, including drug delivery, biomaterials production, gene therapy, new drug synthesis, and nanotechnology (Hikmawanti et al., 2021).

The combination of menthol and thymol into a single NADES (MT NADES) yields a novel, stable liquid solvent at room temperature with enhanced miscibility and potential for pharmaceutical innovation. Notably, this eutectic system exhibits substantially lower volatility than its components, enabling sustained and consistent concentrations of bioactive agents during application. This property enhances the formulation stability and utility of MT NADES in pharmaceutical and biomedical contexts by minimizing compound loss through evaporation or degradation (Aroso et al., 2016; Bergua et al., 2022).

The synergistic antibacterial activity of the menthol–thymol eutectic system has been previously reported (Syed et al., 2022). This observation opens promising avenues for the application of MT NADES in antifungal strategies, particularly for the treatment of *C. albicans* strains exhibiting resistance to conventional therapies.

Reactive oxygen species (ROS) generation is another mechanism involved in the activity of many antifungal agents. ROS is a collective term that includes oxygen radicals and certain non-radicals, oxidizing agents, and/or those easily converted into superoxides, peroxides, and hydroxyl radicals. The alteration of normal levels of ROS induces oxidative damage to biomolecules such as lipids, proteins, and DNA, producing alterations in their structure and thereby threatening cell integrity (Bustos et al., 2016; Lee and Lee, 2018). Although ROS induction has been reported individually for Thy and Men, further studies are needed to elucidate whether the MT NADES system specifically contributes to ROS generation in *C. albicans*.

This study aimed to evaluate the antifungal and fungicidal properties of menthol–thymol (1:1) NADES against a clinical azole-resistant *C. albicans* strain (RCa). Additionally, we investigated the potential oxidative stress induced by MT NADES through ROS production, its capacity to inhibit efflux pumps, and its synergistic interaction with fluconazole.

## 2 Materials and methods

### 2.1 Chemicals

Menthol (Men) (99.79%) was purchased from Parafarm (Argentina). Thymol (Thy) (99%) was purchased from Alkemit (Argentina). Sabouraud glucose broth (SGB) and Sabouraud dextrose agar (SDA) were purchased from Laboratorios Britania S.A., Buenos Aires, Argentina, and were used for antimicrobial assays. Fluconazole (FLZ) (≥98%), tacrolimus (European Pharmacopoeia Reference Standard), and Nile red (NR) suitable for microscopy were purchased from Sigma-Aldrich (St. Louis, USA).

### 2.2 Preparation and characterization of natural deep eutectic systems

The NADES was prepared using Men and Thy in a 1:1 M ratio. The individual components were weighed and introduced into a glass container and then heated and stirred as per Dai et al. (2013) to obtain a stable liquid phase over time. It was then heated to 45 °C under magnetic stirring at 350 rpm until a transparent fluid liquid was formed (approximately 30–90 min). Our group has previously reported the physicochemical and spectrophotometric characteristics of MT NADES: density of 0.9030 g mL^−1^; operational pH of 6.1 ± 0.5; polarity, expressed as transition energy of Nile red (EENR), of 51.06 kcal mol^−1^, being less polar than water (47.9 kcal mol^−1^). Moreover, the formation of hydrogen bonds between the components of the hydrophobic natural deep eutectic system (HPB-NADES) was confirmed by Fourier-transformed infrared spectroscopy with an attenuated total reflectance accessory (FTIR-ATR) (Mammana et al., 2023). The MT NADES remained stable throughout all experiments, showing no signs of phase separation, crystallization, or degradation. Its physical appearance and biological activity were consistent across replicates, confirming system integrity.

### 2.3 Antifungal evaluation

#### 2.3.1 Fungal strains

The RCa (12.99) strain overexpresses CDR1-, CDR2-, and MDR1-like genes (White et al., 2002). These genes are involved in MDR. The strain was isolated from the oral cavity and was kindly provided by Dr. T. White (University of Washington, Seattle, USA). It was grown in yeast peptone dextrose (YPD) broth and stored as frozen stocks with 15% glycerol at −80 °C. Before each experiment, cells were subcultured from this stock onto Sabouraud dextrose agar (Britania S.A., Bs. As. Argentina) (Peralta et al., 2015).

#### 2.3.2 Minimum inhibitory concentration assay

The microdilution method in a 96-well plate was used to determine the minimum inhibitory concentration (MIC), following the standardized microdilution protocol in a 96-well plate, M27-A4 of the CLSI, with some modifications (CLSI, 2017) [18]. An initial inoculum of 10^3^ colony-forming units per mL (CFU/mL) was grown in SGB. Dilutions of MT NADES were prepared at concentrations of 90, 180, and 360 μg/mL. Dilutions of Men and Thy were prepared at concentrations of 15, 30, 60, 100, 200, 500, and 1000 μg/mL. The test was carried out in a 96-well plate by adding 100 µL of the different antifungal solution dilutions and 100 µL of the fungal inoculum with a concentration of 10^3^ CFU/mL. The plate was incubated for 24 h at 36 °C in a culture oven (MARNE Mod. 644C).

The samples were compared with the respective control containing only the SGB. Absorbance was measured at 540 nm using a MicroQuant microplate spectrophotometer (Tecan Sunrise Model, Tecan, Austria).

The MIC values of MT NADES and the compounds Men and Thy were defined as the lowest concentration that produces an optical density of 50% or less relative to the growth control measured at 540 nm in a microplate reader.

#### 2.3.3 Colony-forming unit assays

In brief, 20 mL of Sabouraud dextrose agar was dispensed into sterile 180 × 11 mm Petri dishes and allowed to solidify. A microdilution assay, as previously described for MIC determination, was employed to determine fungal viability. *C. albicans* cells were treated with various concentrations of MT NADES and incubated at 36 °C for 24 h. Samples were collected and serially diluted (10^−4^, 10^−5^, and 10^−6^) with SGB. Then, 10 µL droplets of each dilution were placed on SDA plates. They were prepared in triplicate, and once sown, waited 15 min for the drop to be absorbed; they were then incubated inverted for 24 h at 36 °C. After this incubation period, the colonies were counted, and the resulting number was multiplied by the dilution factor. The number of viable cells was expressed as colony-forming units per milliliter (CFU/mL), calculated as the average of three independent experiments, performed in triplicate.

#### 2.3.4 Intracellular ROS measurement in the RCa strain

A 500 µL aliquot of MT NADES (final concentration 180 μg/mL) or PBS was incubated with 500 µL of cell suspension (1 × 10^6^ cells/mL) at 37 °C for 2 h. Following incubation, the tubes were centrifuged at 12,000 rpm for 5 min. The resulting pellets were resuspended in 400 µL of PBS containing 10 µM H_2_-DCFDA and incubated for 1 h at 37 °C, protected from light. After incubation, samples were centrifuged and washed with 220 µL of PBS. Intracellular ROS were detected using a Synergy HT Multi-Mode Microplate Reader (BioTek Instruments, Inc., USA) fluorometer at excitation/emission wavelengths of 485/528 nm. Fluorescence measurements were taken over 30 min, with background fluorescence corrected using parallel blanks. All experiments were performed in triplicate, with each experimental condition tested in duplicate. The standard error of the mean was calculated based on the triplicate data. The data obtained were graphed using GraphPad software version 8.0.1 (GraphPad Software, San Diego, USA).

#### 2.3.5 Efflux pump activity evaluation by flow cytometry

Efflux pump activity in the RCa was evaluated using a NR accumulation assay, following the protocol described by Iyer et al. (2020) with minor modifications. Tacrolimus was used as a positive control due to its well-established role as an inhibitor of ABC-type efflux pumps in *C. albicans*, enhancing intracellular drug accumulation and reversing resistance to azoles. The cells were cultured overnight in SGB and harvested at mid-log phase. After washing with PBS, they were incubated with MT NADES (all at sub-inhibitory concentrations, 1/2 MIC) or the reference efflux pump inhibitor tacrolimus (100 µM) for 30 min at 30 °C. Nile red (7.5 µM) was added, and samples were incubated in the dark for 10 min. Fluorescence was measured using a Life Technologies Attune NxT flow cytometer (Thermo Fisher Scientific, USA) with excitation/emission settings of 488/585 nm. A total of 40,000 events were recorded per sample. Histograms were generated to evaluate shifts in fluorescence intensity, indicating intracellular dye accumulation due to efflux pump inhibition.

Flow cytometry data were acquired using Attune Cytometric Data Analysis Software and exported in FCS format. Histogram and dot-plot analyses were performed using FlowJo™ v10.8 (BD Biosciences, USA), enabling visualization of fluorescence intensity distributions and population gating. Relative fluorescence units (RFU) for each condition were calculated to quantify NR retention. Comparative statistical analyses were conducted using GraphPad Prism version 8.0.1 (GraphPad Software, San Diego, CA, USA). Duplicates for each experimental condition were included in all experiments.

Data are reported as mean ± standard error of the mean (SEM) from at least three independent replicates. One-way ANOVA followed by Tukey’s multiple comparison test was applied to assess statistical significance. Differences were considered significant at *p* ≤ 0.05.

#### 2.3.6 Checkerboard microtiter plate testing

The checkerboard method was chosen to determine the possible interactions between MT NADES and FLZ in RCa cell growth. It was carried out according to the standardized protocol M27-A4 of the CLSI (CLSI, 2017). Rows A–H of the 96-well plate contained increasing concentrations of MT NADES (25 μg/mL to 400 μg/mL). Each subsequent row contained double the concentrations of the previous row. The same procedure was carried out along the columns (1–12) with FLZ at concentrations ranging from 0.125 μg/mL to 64 μg/mL. On the same plate, the respective growth controls of the RCa strain were carried out in the absence of the compounds. Thus, each well contained a single combination of MT NADES and FLZ (Iten et al., 2009). The compounds were diluted with SGB from a stock solution. Subsequently, 100 µL of an initial inoculum of 10^3^ CFU/mL of RCa was added. The plate was incubated at 36 °C for 24 h. Absorbance was measured at 540 nm using a MicroQuant microplate spectrophotometer (Tecan Sunrise Model, Tecan, Austria).

The MIC, fractional inhibitory concentration (FIC), and fractional inhibitory concentration index (FICI) values were calculated. The MIC for compounds was defined as the lowest concentration producing an optical density of 50% or less relative to the growth control measured at 540 nm in a microplate reader. The FIC was calculated as the MIC of MT NADES and FLZ when combined and divided by the MIC of the compound alone. FICI was the sum of the FIC of each compound (CLSI, 2017).

The data obtained were graphed using GraphPad software version 8.0.1 (GraphPad Software, San Diego, USA), and the standard error of three independent experiments was calculated. Duplicates for each experimental condition were included in all experiments.

Statistical analysis was performed using the one-way ANOVA test and Tukey’s test. *p* ≤ 0.05 represented a significant difference between the groups. Data were expressed as the mean ± standard error of the mean (SEM).

#### 2.3.7 Disc diffusion assay

The disk diffusion assay test followed the CLSI recommendations document M44-A2 with some modifications (CLSI, 2009). In brief, Sabouraud dextrose agar was prepared, and this culture medium was sterilized and incorporated into Petri dishes (10 cm). After cooling, the plates were inoculated with the *C. albicans* suspension adjusted to a 0.5 McFarland concentration.

Likewise, sterile discs were impregnated with 360 µg of MT NADES, 24 µg of FLZ, and their combination, MT NADES + FLZ, at half the concentration at which each compound was evaluated separately—that is, 190 µg of MT NADES + 12 µg of FLZ. The discs were placed on the plates previously inoculated with the strain, and the plates were incubated at 36 °C for 24 h.

After this period, the plates were photographed, and the antimicrobial activity of the compounds was assessed by measuring the diameter of the microbial growth inhibition zone in millimeters (mm).

## 3 Results

### 3.1 The minimum inhibitory concentration of MT NADES against azole-resistant *C. albicans*

The antifungal activity of MT NADES was evaluated using a clinical strain of RCa. The MIC values were determined using the standardized CLSI microdilution protocol (M27-A4) with modifications described by Peralta et al. (2012) and CLSI (2017). The MIC values of MT NADES against the RCa strain were 180 μg/mL, achieving 56.19% inhibition relative to the control grown in SDB alone. At a higher concentration of 360 μg/mL, MT NADES exhibited an even greater inhibition effect on RCa growth (90.77%).

The Men MIC was determined at 1000 μg/mL, while the Thy MIC value was 200 μg/mL for the RCa strain, with a growth inhibition percentage of 67% and 60%, respectively.

### 3.2 Colony-forming unit assays

**FIGURE 2:**
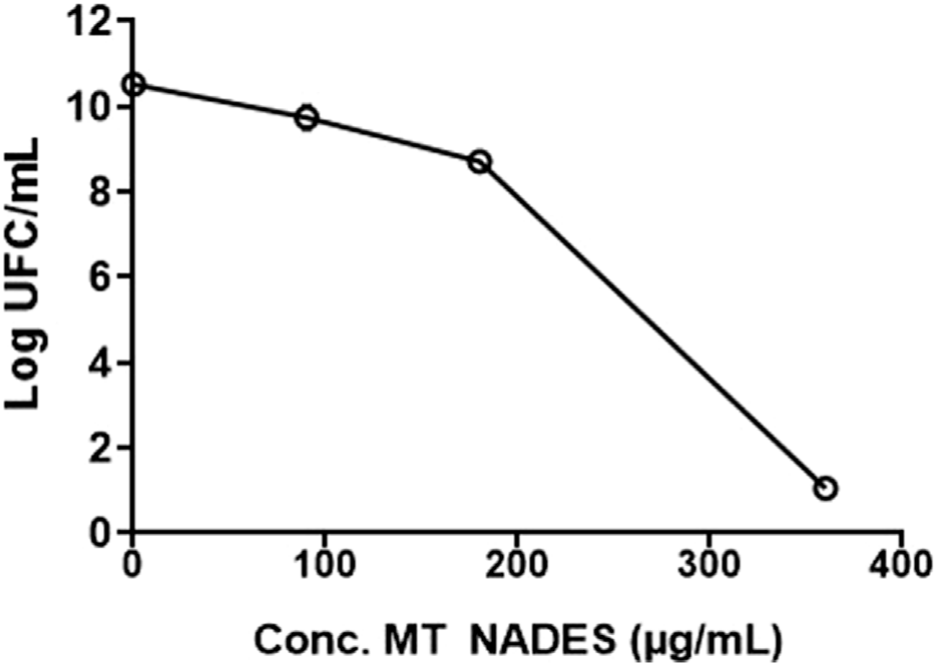
Cell viability of the azole-resistant *C. albicans* strain (RCa) in the presence of MT NADES at different concentrations.

Given the observed antifungal activity, the viability of the RCa strain was evaluated in the presence of different concentrations of MT NADES (90, 180, and 360 μg/mL) by performing colony counts using the microdrop technique. Similar to *C. albicans* growth, MT NADES significantly reduced cell viability. In fact, after 24 h of incubation with 180 μg/mL of MT NADES, the percentage reduction in survival values compared to the control was 98.9%. At 360 μg/mL of MT NADES, a complete fungicidal effect was observed, with no CFU growth on the plate (Figure 2). The MFC/MIC ratio for MT NADES was 2, indicating its fungicidal effect of the MT NADES, as a ratio less than 4 signifies fungicidal activity (Klepser et al., 1997; Pfaller et al., 2004; Meletiadis et al., 2007).

### 3.3 Intracellular ROS measurement in the RCa strain

**FIGURE 3:**
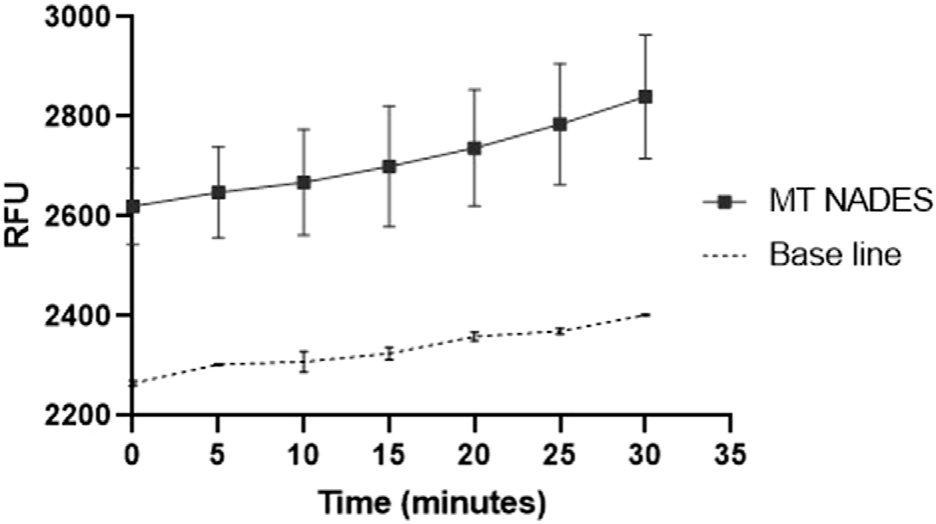
ROS production by the azole-resistant *C. albicans* strain (RCa) in the presence of MT NADES at 180 μg/mL. The figure shows the production of ROS expressed in relative fluorescence units (RFU) as a function of time. The control curve corresponds to cells in the culture medium.

ROS production by the RCa in the presence of 180 μg/mL of MT NADES was assessed (Figure 3). The results are presented as RFU over time. The control group, consisting of cells incubated in a culture medium alone, exhibited baseline levels of ROS production. In contrast, cells treated with MT NADES demonstrated a significant increase in ROS levels, particularly after the initial 10 min, indicating a strong oxidative response, reaching an 18% increase in ROS after 30 min of measurement (Figure 3).

### 3.4 Efflux pump inhibition in azole-resistant *C. albicans* by menthol–thymol NADES assessed via Nile red fluorescence

To evaluate whether MT NADES modulates efflux pump activity, a functional assay was performed using NR accumulation in the RCa. After treatment with MT NADES at sub-inhibitory concentrations (90 μg/mL, equivalent to ½ MIC), RCa cells exhibited a markedly increased intracellular fluorescence intensity compared to untreated controls, indicating reduced efflux activity.

**FIGURE 4:**
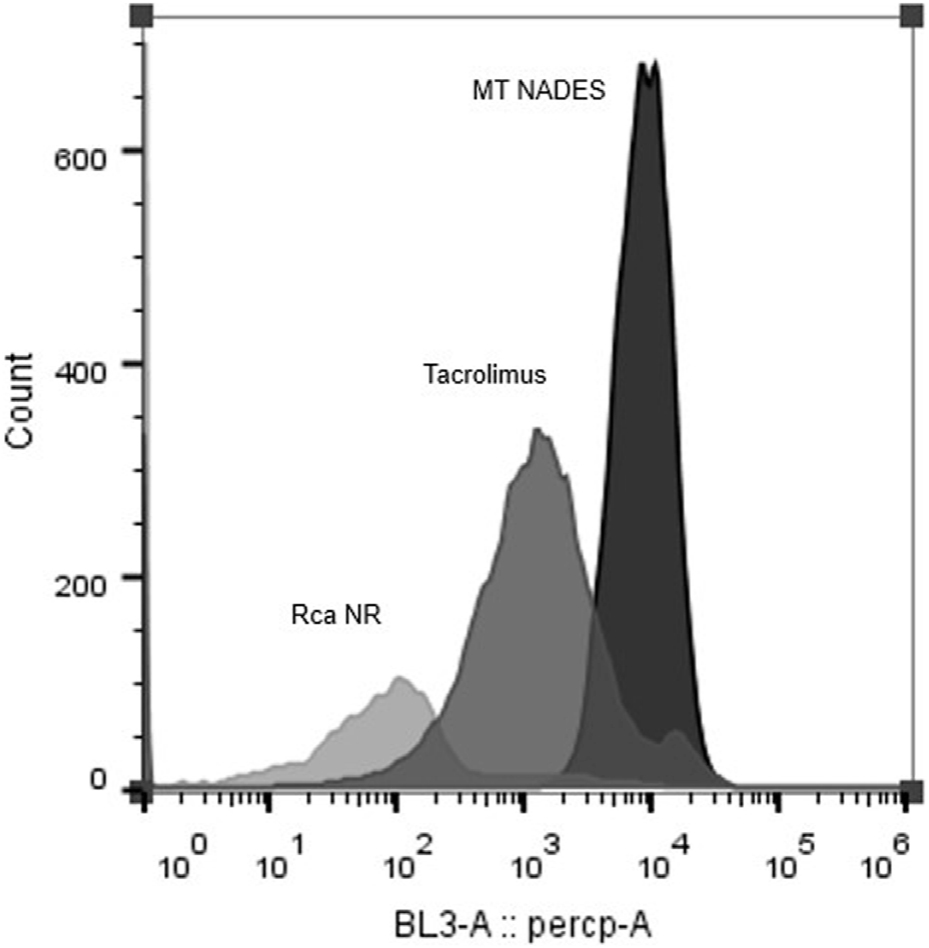
Flow cytometry fluorescence histograms of Nile red-stained azole-resistant *C. albicans* (RCa) following treatment with MT NADES (90 μg/mL) and tacrolimus (100 µM). A pronounced rightward shift in fluorescence intensity is observed in the presence of MT NADES. This indicates greater intracellular dye retention and suggests stronger efflux pump inhibition than the reference inhibitor tacrolimus.

**FIGURE 5:**
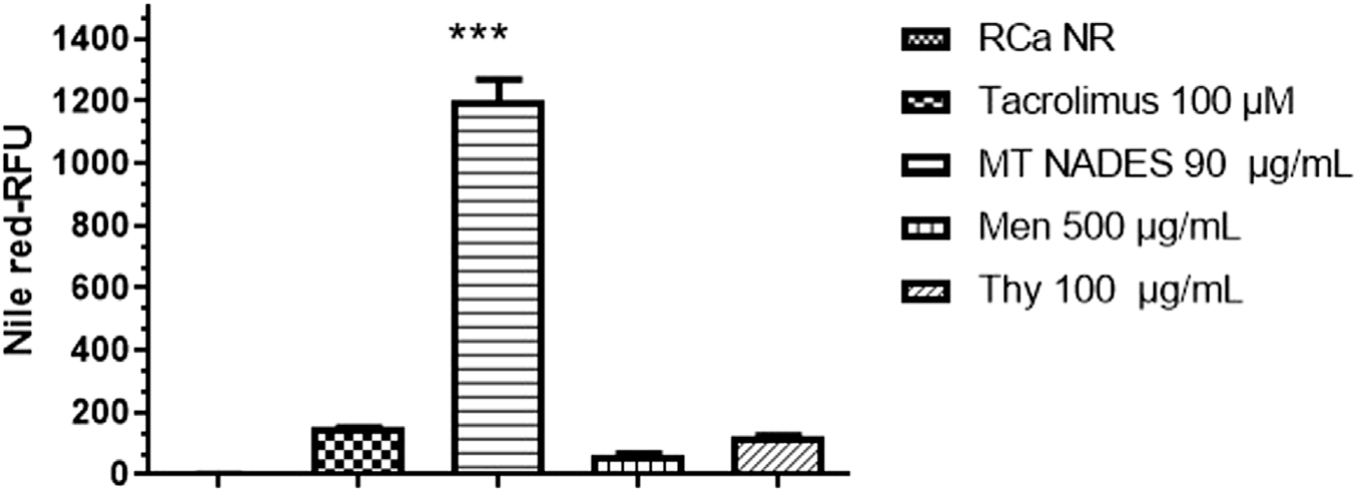
Nile red retention in azole-resistant *C. albicans* (RCa) following treatment with menthol (Men), thymol (Thy), the menthol–thymol NADES (MT NADES) (all at 1/2 MIC), and tacrolimus (100 µM). RFU of untreated RCa stained with Nile red (RCa NR) were set as the baseline (1.0 arbitrary unit) after autofluorescence subtraction. MT NADES induced the highest dye retention, surpassing both individual components and the reference inhibitor, indicating a potent inhibition of efflux pump activity. Data represent mean ± SD (*n* = 3). ***p < 0.0001 denotes statistically significant difference between MT NADES (90 μg/mL) and tacrolimus (100 µM), Men (500 μg/mL), and Thy (100 μg/mL).

Flow cytometry histograms clearly show a fluorescence shift toward higher values in MT NADES-treated cells, indicating enhanced NR retention (Figure 4). This shift was significantly greater than that observed with the reference efflux pump inhibitor tacrolimus at 100 µM. Quantitative analysis revealed that MT NADES induced a 10-fold increase in NR retention compared to tacrolimus (Figure 5).

Furthermore, as shown in Figure 5, the relative retention levels for menthol, thymol, MT NADES (each at 1/2 MIC), and tacrolimus (100 µM) demonstrate differential inhibitory capacities. Menthol increased dye retention to approximately 50% of that observed with tacrolimus, while thymol exhibited a similar effect to tacrolimus. In contrast, the full MT NADES system produced a substantially stronger effect, confirming its superior ability to impair efflux pump function in resistant *C. albicans*.

These results suggest that the chemosensitizing activity of MT NADES involves not only ROS induction but also a significant inhibition of efflux transporters such as Cdr1p, Cdr2p, and Mdr1p, positioning MT NADES as a promising multifunctional antifungal agent.

### 3.5 Checkerboard microdilution assay

Given the significant fungicidal activity of MT NADES against the azole-resistant RCa strain with MDR, our next objective was to investigate the interactions of MT NADES in combination with the reference antifungal, fluconazole (FLZ). The MIC obtained for MT NADES, FLZ, and their combination for the RCa strain is detailed in Table 1.

**TABLE 1:** MIC, FIC, and FICI values of MT NADES and FLZ alone and in combination for the RCa strain.

Treatment	MIC alone (µg/mL)	MIC combination (µg/mL)	FIC	FICI
MT NADES	180	50	0.28	0.2839
FLZ	32	0.125	0.0039	

**FIGURE 6:**
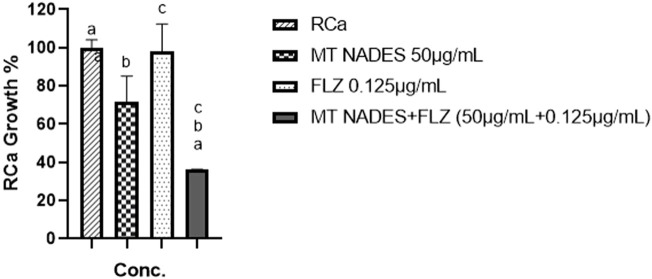
Percentage of growth of azole-resistant *C. albicans* (RCa) following treatment with menthol–thymol NADES (MT NADES, 50 μg/mL), fluconazole (FLZ, 0.125 μg/mL), and their combination (MT NADES + FLZ). The combined treatment significantly reduced fungal growth compared to the untreated control and to each compound alone at the same concentrations, indicating a synergistic antifungal effect. ^a^ *p* = 0.0067 denotes significant difference between RCa strain and MT NADES + FLZ (50 μg/mL + 0.125 μg/mL). ^b^ *p* = 0.0324 denotes significant difference between MT 50 μg/mL and MT NADES + FLZ (50 μg/mL + 0.125 μg/mL). ^c^ *p* = 0.0075 denotes significant difference between FLZ 0.125 μg/mL and MT NADES + FLZ (50 μg/mL + 0.125 μg/mL).

By combining MT NADES with FLZ, the MIC of MT NADES (180 μg/mL) was reduced fourfold, while for FLZ, the MIC (32 μg/mL) decreased 256-fold, achieving the chemosensitization of RCa to FLZ. The proportion of RCa growth in the presence of MT NADES and FLZ at the MIC of their combination was 1/4 MIC for MT NADES and 1/256 for FLZ (Figure 6). Using the checkerboard model, the FIC for MT NADES and FLZ was determined as follows: 0.28 and 0.0039, respectively, with an FICI of 0.2839. The interpretation of the FICI was as suggested by Odds (2003): synergy (FICI ≤ 0.5), antagonism (FICI > 4.0), and no interaction (FICI > 0.5–4.0). The FICI value (less than 0.5) obtained for the combination suggests a synergistic interaction between MT NADES and FLZ (Odds, 2003).

Figure 6 compares the effects of MT NADES at its FIC (50 μg/mL), FLZ at its FIC (0.125 μg/mL), and their combination. The combined treatment significantly reduces RCa growth (63.89%) compared to the control and the individual effects of each compound at the same concentrations.

### 3.6 Disc diffusion assay

The determination of susceptibility by the disc diffusion method denoted the resistance of RCa to FLZ (24 µg) according to the breakpoints indicated in document M44-A2 of the CLSI, with an inhibition halo of 10 mm (less than 14 mm) at the mentioned concentration (CLSI, 2009).

**FIGURE 7:**
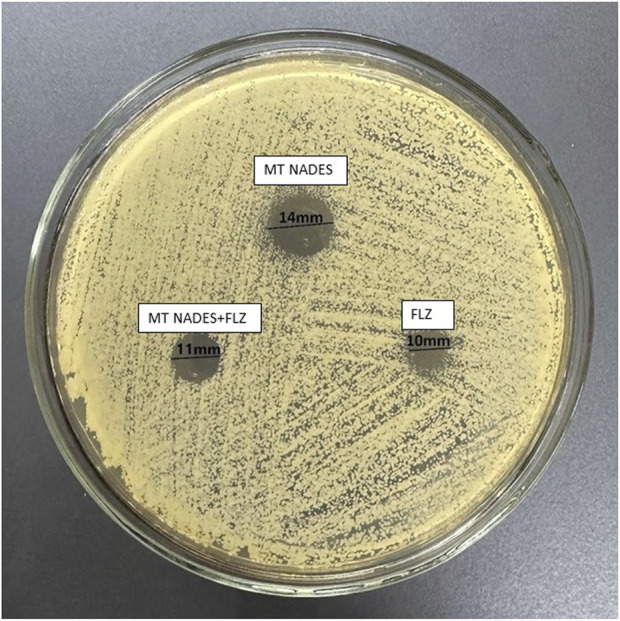
Azole-resistant *C. albicans* (RCa) sensitivity was determined using the disc diffusion method. MT NADES (menthol–thymol NADES), FLZ (fluconazole), and MT NADES + FLZ (menthol–thymol NADES + fluconazole).

Table 2 details the values obtained from the inhibition zones of MT NADES at 360 μg, FLZ at 24 μg, and their combination at half the concentration of each compound, expressed in mm ± standard deviation (SD) (Figure 7).

**TABLE 2:** Inhibition zones of MT NADES, FLZ, and their combination, expressed in mm ± SD.

Treatment	Concentration (µg/mL)	Inhibition zone diameter (mm) mean ± SD
MT NADES	360	14 ± 1
FLZ	24	10 ± 1
MT NADES + FLZ	180 + 12	11 ± 1.5

## 4 Discussion

The antifungal efficacy of MT NADES against the RCa highlights the potential of sustainable natural solvents in combating MDR pathogens. The MIC and MFC values of 180 and 360 μg/mL, respectively, demonstrate MT’s potent fungicidal activity, with an MFC/MIC ratio of 2, confirming its classification as fungicidal (Klepser et al., 1997; Pfaller et al., 2004).

This activity significantly reduces the growth and viability of RCa, as evidenced by a 98.9% reduction in CFU counts at the MIC and complete inhibition at the MFC. To connect and contextualize our results with findings from other authors on menthol-based deep eutectic solvents (DESs), it is important to highlight how the unique properties of MT NADES align with the broader research on the applications and mechanisms of such solvents (Cherniakova et al., 2024).

The observed increase in ROS production in the presence of MT NADES strongly supports oxidative stress as a key mechanism underlying its antifungal activity. ROS generation has been widely associated with disrupting fungal cell structures, including membranes, vacuoles, and microtubules, ultimately leading to cell death (Cherniakova et al., 2024). This mechanism is consistent with the effects of other terpene-based compounds such as eugenol, citral, and 1,8-cineole, which are essential oil components known to induce ROS-mediated oxidative stress in *C. albicans* (Shahina et al., 2022b; 2022a).

Our findings reveal that MT NADES, containing Men and Thy, similarly triggers significant ROS production, paralleling the oxidative effects observed with eugenol. This increase in ROS may be linked to the disruption of mitochondrial membrane potential and respiratory chain function, a mechanism previously described for other terpenes such as carvacrol and thymol. In *C. albicans*, these compounds have been shown to interfere with mitochondrial respiration, leading to ROS leakage and oxidative damage to cellular components (Shahina et al., 2022a). While no studies to date have specifically reported pro-oxidative effects of NADES in fungal cells, the increase in ROS observed in our assays suggests that MT NADES may share a similar mitochondria-mediated mechanism of action.

These results not only reinforce the hypothesis that ROS generation is a critical driver of antifungal activity in terpene-based compounds but also highlight the capacity of the deep eutectic system MT NADES to induce cellular stress as a prominent mechanism of action.

Further investigation into the interplay between ROS levels and cellular damage across diverse antifungal agents, including MT NADES, could provide valuable insights into optimizing their therapeutic applications and understanding their role in combating fungal resistance.

A critical finding of our study is the capacity of MT NADES to inhibit efflux pump activity in a resistant *C. albicans* strain overexpressing Cdr1p, Cdr2p, and Mdr1p transporters. This activity was evidenced by the increased intracellular accumulation of NR, a known substrate of MDR transporters. Remarkably, MT NADES at sub-inhibitory concentrations (1/2 MIC) enhanced dye retention 10-fold more than tacrolimus, a well-established inhibitor of ABC transporters. This suggests that MT NADES exerts a strong inhibitory effect on key efflux pumps such as Cdr1p, Cdr2p, and Mdr1p, which are often overexpressed in resistant *C. albicans* strains and are pivotal to azole resistance. These results are in line with studies showing that certain natural compounds, including terpenes, can disrupt MDR mechanisms by targeting these transport systems (Ahmad et al., 2013; Muslim and Hussin, 2020).

By impairing efflux pump function, MT NADES may increase the intracellular accumulation of fluconazole and other antifungals, thus enhancing their activity even in resistant strains. This mechanism likely contributes to the remarkable chemosensitizing effect observed in our checkerboard assays, where fluconazole’s MIC was reduced 256-fold in the presence of MT NADES. Such synergistic interactions are highly valuable in the fight against MDR fungi (Engle and Kumar, 2024; Jaiswal and Kumar, 2024) as they not only restore the efficacy of existing drugs but also allow for lower therapeutic doses, potentially reducing toxicity. Moreover, the dual action of MT NADES, through both ROS generation and efflux pump inhibition, positions this NADES as a promising adjuvant strategy to counteract antifungal resistance. The ability of natural eutectic systems to modulate cellular resistance pathways warrants further exploration in antifungal drug development (Engle and Kumar, 2024; Jaiswal and Kumar, 2024).

We emphasize that menthol-based DESs are hydrophobic solvents with tunable properties, making them highly effective for extracting bioactive compounds and enhancing their bioavailability. These systems are particularly noted for their role in stabilizing volatile and reactive terpenes, addressing challenges such as oxidation and decomposition, which are common in natural product research (Cherniakova et al., 2024).

Our study demonstrates how these stabilized systems can extend beyond extraction to exert direct antifungal effects. This aligns with the findings of Cherniakova et al. (2024) that menthol-based DESs can influence the solubility and interaction of active compounds with biological membranes, which potentially explains the fungicidal properties observed in our research.

From a pharmaceutical perspective, formulating menthol and thymol as a NADES enhances their biological activity and confers significant physicochemical advantages. Notably, MT NADES exhibits markedly reduced volatility than its components, resulting in more stable concentrations at the application site. This property is particularly advantageous for topical or mucosal antifungal formulations as it minimizes compound loss due to evaporation and may contribute to prolonged local bioavailability and sustained antifungal action. Furthermore, the stable liquid state of MT NADES at room temperature facilitates consistent bioactive exposure and reduces the risk of compound degradation under standard conditions. These characteristics, combined with its potent biological effects and eco-friendly profile (Mammana et al., 2023), reinforce the potential of MT NADES as a multifunctional platform for antifungal drug development, especially in the context of multidrug-resistant *C. albicans*.

## 5 Conclusion

Our investigation expands the applications of menthol-and-thymol-based NADES beyond extraction, displaying their dual role as eco-friendly solvents and bioactive agents *per se*. This connection underscores the versatility and therapeutic potential of these systems. Further exploration of their properties in other biological contexts could provide deeper insights into their full capabilities.

The fungicidal properties of MT NADES, ROS-mediated mechanism, and synergistic interaction with fluconazole make it a promising candidate for addressing MDR fungal infections. Future studies should explore its clinical applications, stability, and potential for broader antifungal activity *in vivo*, advancing the sustainable use of natural products in antifungal therapy.

## Data Availability

The raw data supporting the conclusions of this article will be made available by the authors, without undue reservation.

## Abbreviations

ABC, ATP-binding cassette; ANOVA, analysis of variance; CFU, colony-forming units; CLSI, Clinical and Laboratory Standards Institute; FIC, fractional inhibitory concentration; FICI, fractional inhibitory concentration index; FTIR-ATR, Fourier-transformed infrared spectroscopy with an attenuated total reflectance accessory; MDR, multidrug resistance; Men, menthol; MFS, major facilitator superfamily; MIC, minimum inhibitory concentration; NR, Nile red; RCa, resistant *Candida albicans*; RFU, relative fluorescence units; ROS, reactive oxygen species, SDA, Sabouraud dextrose agar; SEM, standard error of the mean; SGB, Sabouraud glucose broth; Thy, thymol; YPD, yeast extract peptone dextrose; WHO, World Health Organization.

## References

[B1] Ahmad A. Khan A. Manzoor N. (2013). Reversal of efflux mediated antifungal resistance underlies synergistic activity of two monoterpenes with fluconazole. Eur. J. Pharm. Sci. 48, 80–86. 10.1016/j.ejps.2012.09.016 23111348

[B2] Aroso I. M. Silva J. C. Mano F. Ferreira A. S. D. Dionísio M. Sá-Nogueira I. (2016). Dissolution enhancement of active pharmaceutical ingredients by therapeutic deep eutectic systems. Eur. J. Pharm. Biopharm. 98, 57–66. 10.1016/j.ejpb.2015.11.002 26586342

[B3] Bergua F. Castro M. Lafuente C. Artal M. (2022). Thymol+l-menthol eutectic mixtures: thermophysical properties and possible applications as decontaminants. J. Mol. Liq. 368, 120789. 10.1016/j.molliq.2022.120789

[B4] Bustos P. S. Deza-Ponzio R. Páez P. L. Albesa I. Cabrera J. L. Virgolini M. B. (2016). Protective effect of quercetin in gentamicin-induced oxidative stress *in vitro* and *in vivo* in blood cells. Effect on gentamicin antimicrobial activity. Environ. Toxicol. Pharmacol. 48, 253–264. 10.1016/j.etap.2016.11.004 27846408

[B5] Cherniakova M. Varchenko V. Belikov K. (2024). Menthol‐based (deep) eutectic solvents: a review on properties and application in extraction. Chem. Rec. 24. 10.1002/tcr.202300267 37861277

[B6] CLSI (2009). Method for antifungal disk diffusion susceptibility testing of yeasts.

[B7] CLSI (2017). M27-A4: reference method for broth dilution antifungal susceptibility testing of yeasts. Available online at: https://clsi.org/standards/products/microbiology/documents/m27/.

[B8] Cushnie T. P. T. Cushnie B. Echeverría J. Fowsantear W. Thammawat S. Dodgson J. L. A. (2020). Bioprospecting for antibacterial drugs: a multidisciplinary perspective on natural product source material, bioassay selection and avoidable pitfalls. Pharm. Res. 37, 125. 10.1007/s11095-020-02849-1 32529587

[B9] Dai Y. van Spronsen J. Witkamp G.-J. Verpoorte R. Choi Y. H. (2013). Natural deep eutectic solvents as new potential media for green technology. Anal. Chim. Acta 766, 61–68. 10.1016/j.aca.2012.12.019 23427801

[B10] Engle K. Kumar G. (2024). Tackling multi-drug resistant fungi by efflux pump inhibitors. Biochem. Pharmacol. 226, 116400. 10.1016/j.bcp.2024.116400 38945275

[B11] Espino M. Solari M. Fernández M. de los Á. Boiteux J. Gómez M. R. Silva M. F. (2019). NADES-Mediated folk plant extracts as novel antifungal agents against Candida albicans. J. Pharm. Biomed. Anal. 167, 15–20. 10.1016/j.jpba.2019.01.026 30738239

[B12] Garvey M. Meade E. Rowan N. J. (2022). Effectiveness of front line and emerging fungal disease prevention and control interventions and opportunities to address appropriate eco-sustainable solutions. Sci. Total Environ. 851, 158284. 10.1016/j.scitotenv.2022.158284 36029815

[B13] Hikmawanti N. P. E. Ramadon D. Jantan I. Mun’im A. (2021). Natural deep eutectic solvents (NADES): phytochemical extraction performance enhancer for pharmaceutical and nutraceutical product development. Plants 10, 2091. 10.3390/plants10102091 34685899 PMC8538609

[B14] Iten F. Saller R. Abel G. Reichling J. (2009). Additive antimicrobial [corrected] effects of the active components of the essential oil of thymus vulgaris--chemotype carvacrol. Planta Med. 75, 1231–1236. 10.1055/s-0029-1185541 19347798

[B15] Iyer K. R. Robbins N. Cowen L. E. (2020). Flow cytometric measurement of efflux in Candida species. Curr. Protoc. Microbiol. 59, e121. 10.1002/cpmc.121 33047867 PMC8515609

[B16] Jaiswal N. Kumar A. (2024). Modulators of Candida albicans membrane drug transporters: a lucrative portfolio for the development of effective antifungals. Mol. Biotechnol. 66, 960–974. 10.1007/s12033-023-01017-1 38206530

[B17] Klepser M. E. Wolfe E. J. Jones R. N. Nightingale C. H. Pfaller M. A. (1997). Antifungal pharmacodynamic characteristics of fluconazole and amphotericin B tested against Candida albicans. Antimicrob. Agents Chemother. 41, 1392–1395. 10.1128/AAC.41.6.1392 9174207 PMC163923

[B18] Lee W. Lee D. G. (2018). Reactive oxygen species modulate itraconazole-induced apoptosis *via* mitochondrial disruption in Candida albicans. Free Radic. Res. 52, 39–50. 10.1080/10715762.2017.1407412 29157011

[B19] Mammana S. B. Jofré M. F. Cohen A. C. Gómez F. J. V. Silva M. F. (2023). Selective extraction and preconcentration of melatonin mediated by hydrophobic natural deep eutectic systems. Microchem. J. 194, 109317. 10.1016/j.microc.2023.109317

[B20] Meletiadis J. Antachopoulos C. Stergiopoulou T. Pournaras S. Roilides E. Walsh T. J. (2007). Differential fungicidal activities of Amphotericin B and voriconazole against aspergillus species determined by microbroth methodology. Antimicrob. Agents Chemother. 51, 3329–3337. 10.1128/AAC.00345-07 17576838 PMC2043246

[B21] Muslim S. N. Hussin Z. S. (2020). Chemical compounds and synergistic antifungal properties of thymus kotschanus essential oil plus ketoconazole against Candida spp. Gene Rep. 21, 100916. 10.1016/j.genrep.2020.100916

[B22] Odds F. C. (2003). Synergy, antagonism, and what the chequerboard puts between them. J. Antimicrob. Chemother. 52, 1. 10.1093/jac/dkg301 12805255

[B23] Peralta M. Calise M. Fornari M. Ortega M. Diez R. Cabrera J. (2012). A prenylated flavanone from Dalea elegans inhibits rhodamine 6 G efflux and reverses fluconazole-resistance in Candida albicans. Planta Med. 78, 981–987. 10.1055/s-0031-1298627 22673834

[B24] Peralta M. A. da Silva M. A. Ortega M. G. Cabrera J. L. Paraje M. G. (2015). Antifungal activity of a prenylated flavonoid from Dalea elegans against Candida albicans biofilms. Phytomedicine 22, 975–980. 10.1016/j.phymed.2015.07.003 26407939

[B25] Pfaller M. A. Sheehan D. J. Rex J. H. (2004). Determination of fungicidal activities against yeasts and molds: lessons learned from bactericidal testing and the need for standardization. Clin. Microbiol. Rev. 17, 268–280. 10.1128/CMR.17.2.268-280.2004 15084501 PMC387411

[B26] Santi M. D. Ortega M. G. Peralta M. A. (2022). A state-of-the-art review and prospective therapeutic applications of Prenyl flavonoids as chemosensitizers against antifungal multidrug resistance in Candida albicans. Curr. Med. Chem. 29, 4251–4281. 10.2174/0929867329666220209103538 35139777

[B27] Shahina Z. Al Homsi R. Price J. D. W. Whiteway M. Sultana T. Dahms T. E. S. (2022a). Rosemary essential oil and its components 1,8-cineole and α-pinene induce ROS-dependent lethality and ROS-independent virulence inhibition in Candida albicans. PLoS One 17, e0277097. 10.1371/journal.pone.0277097 36383525 PMC9668159

[B28] Shahina Z. Ndlovu E. Persaud O. Sultana T. Dahms T. E. S. (2022b). Candida albicans reactive oxygen species (ROS)-dependent lethality and ROS-independent hyphal and biofilm inhibition by eugenol and citral. Microbiol. Spectr. 10. 10.1128/spectrum.03183-22 PMC9769929 36394350

[B29] Silva E. Oliveira F. Silva J. M. Reis R. L. Duarte A. R. C. (2021). Untangling the bioactive properties of therapeutic deep eutectic solvents based on natural terpenes. Curr. Res. Chem. Biol. 1, 100003. 10.1016/j.crchbi.2021.100003

[B30] Syed U. T. Leonardo I. C. Mendoza G. Gaspar F. B. Gámez E. Huertas R. M. (2022). On the role of components of therapeutic hydrophobic deep eutectic solvent-based nanoemulsions sustainably produced by membrane-assisted nanoemulsification for enhanced antimicrobial activity. Sep. Purif. Technol. 285, 120319. 10.1016/j.seppur.2021.120319

[B31] Tan Y. Lin Q. Yao J. Zhang G. Peng X. Tian J. (2023). *In vitro* outcomes of quercetin on Candida albicans planktonic and biofilm cells and *in vivo* effects on vulvovaginal candidiasis. Evidences of its mechanisms of action. Phytomedicine 114, 154800. 10.1016/j.phymed.2023.154800 37043980

[B32] Tanaka N. Kashiwada Y. (2021). Phytochemical studies on traditional herbal medicines based on the ethnopharmacological information obtained by field studies. J. Nat. Med. 75, 762–783. 10.1007/s11418-021-01545-7 34255289 PMC8397699

[B33] White T. C. Holleman S. Dy F. Mirels L. F. Stevens D. A. (2002). Resistance mechanisms in clinical isolates of Candida albicans. Antimicrob. Agents Chemother. 46, 1704–1713. 10.1128/AAC.46.6.1704-1713.2002 12019079 PMC127245

[B34] WHO (2022). WHO fungal priority pathogens list to guide research, development and public health action. Available online at: https://www.who.int/publications/i/item/9789240060241.

